# The Visual Perception of Material Properties Affects Motor Planning in Prehension: An Analysis of Temporal and Spatial Components of Lifting Cups

**DOI:** 10.3389/fpsyg.2020.00215

**Published:** 2020-02-18

**Authors:** Kristín Ósk Ingvarsdóttir, Christian Balkenius

**Affiliations:** Lund University Cognitive Science, Lund University, Lund, Sweden

**Keywords:** motor planning, material properties, motion capture, center of mass, expectations

## Abstract

The current study examined the role of visually perceived material properties in motor planning, where we analyzed the temporal and spatial components of motor movements during a seated reaching task. We recorded hand movements of 14 participants in three dimensions while they lifted and transported paper cups that differed in weight and glossiness. Kinematic- and spatial analysis revealed speed-accuracy trade-offs to depend on visual material properties of the objects, in which participants reached slower and grabbed closer to the center of mass for stimuli that required to be handled with greater precision. We found grasp-preparation during the first encounters with the cups was not only governed by the anticipated weight of the cups, but also by their visual material properties, namely glossiness. After a series of object lifting, the execution of reaching, the grip position, and the transportation of the cups from one location to another were preeminently guided by the object weight. We also found the planning phase in reaching to be guided by the expectation of hardness and surface gloss. The findings promote the role of general knowledge of material properties in reach-to-grasp movements, in which visual material properties are incorporated in the spatio-temporal components.

## Introduction

Previous research on material perception has largely focused on passive viewing of either real-world images or digitally produced images of material properties and classes, in which it has been found that we are relatively fast at recognizing and categorizing materials based on visual appearance alone (Sharan et al., 2009, 2014; Fleming et al., 2013; Fleming, 2014). However, as we move around in our environment actively exploring, we rarely perceive materials in static and passive scenes (Gibson, 1979). Nevertheless, despite the growing interest in material perception as an active process, less is known about how the visual appearance of material properties are incorporated into motor planning. Especially when conducting a seemingly effortless everyday task using familiar object, such as reaching for a cup of coffee in the morning.

One of many challenges in research on visual material perception is to understand how material properties that are intrinsic to an object, and usually perceived through touch, are visually estimated and anticipated. Before lifting an object, we anticipate the required kinematics and force to lift the object using the provided visual information and memory of previous sensorimotor experience of interacting with similar objects. Such anticipation is interesting considering that some material properties are almost exclusively perceived haptically (e.g., weight and hardness) or visually (e.g., color and glossiness) while other properties, such as size and shape, are readily accessible via both sensory systems (Johansson, 1996; Woods and Newell, 2004). Despite the complexity of the provided sensory information we seem to anticipate them effortlessly.

A growing number of studies (Gordon et al., 1991a, b; Johansson, 1996; Flanagan and Beltzner, 2000; Mon-Williams and Murray, 2000; Flanagan et al., 2006, 2008; Cole, 2008; Buckingham et al., 2009, 2016; Baugh et al., 2012) have shown that we are fairly good at anticipating appropriate application of grip-force when reaching for objects, as we are equipped with internal models that allow the grasping to be planned and adjusted accordingly to avoid slippage. These models are long-term priors formed through time with repeated experience of objects having certain visual appearances, weight, and density. Such as a size-weight association, that proposes that larger objects are normally heavier than smaller objects, and a material-density association, that proposes that objects made of materials with high density are heavier than objects with low density.

Due to its tender age as a research field, material perception has been largely studied using passive viewing of images of material properties and classes. Such approach disregards the impact of materials in a real-world setting. In the present study, we therefore examined the role of visual material properties in motor planning when handling familiar objects. In particular, we examined how changes in material surface appearance, and object weight, resulted in changes in motor movements. A seated reaching task was conducted, in which thin-walled paper cups, that varied in weight and glossiness, were grabbed and transported. We used a motion capture technology to accurately record the movement speed and movement position in three dimensions to achieve both temporal and spatial data for our analysis.

The role of object weight has been well-established in reach-to-grasp studies, whereas less is known about how such tactile information is anticipated based on the visual characteristics of the material category. Although few studies have addressed the noteworthy role of perceived surface texture and surface friction in prehension (Fikes et al., 1994; Flatters et al., 2012; Paulun et al., 2016), the investigation of the role of visual material properties in prehension is remarkably little, especially when using familiar objects. Conceptually, surface gloss is perceived visually but it can convey tactile information, such as friction. Adams et al. (2016) demonstrated such cross-modal relationship between surface gloss and perceived friction. Using discrimination paradigm, they presented computer-generated objects to their participants, either visually on a screen or haptically using a Phantom force feedback device, and found that slipperiness had a positive relationship with objects with shiny surfaces. Additionally, longer approaching times have been reported when reaching for slippery objects compared to objects with high friction (e.g., Fikes et al., 1994; Paulun et al., 2016).

On that note, it would be interesting to see if cross-modal relationships can be found for other material properties. In particular if hardness can be anticipated using the object’s surface gloss, given that some objects with glossy appearances happen to be made of hard materials (e.g., plastic mobile cases, cutlery, and laminated paper). Although, Adams et al. (2016) found no correlation between perceived glossiness and physical compliance, and no relationship between physical surface gloss and perceived compliance. It would be interesting to examine if this is the case for real objects made of materials that are familiar to the observer, instead of phantom objects. Considering that both material category and context have plausible roles in anticipating the direction of the relationship between surface gloss and hardness. For instance, melted butter has glossy appearance and soft quality, whereas a glossy snooker ball has hard qualities. In the current study, we wanted to examine if glossiness could convey information about the hardness of the object when interacting with real objects. We used familiar objects made of similar materials, except some of them were altered with layers of varnish to create a glossy appearance and that way a different material appearance. Besides our interest in the role of surface gloss and object weight in prehension, we were also interested in the subjective impression of hardness based on the visual appearance of the objects alone as an additional measurement, to provide new and exciting data about material perception in motor control.

The kinematics of prehension are comprised of reaching and grasping. In reaching the hand is guided to the targeted location, whereas in grasping the observer prepares the grip by opening and closing the hand according to the targeted object’s properties (Jeannerod, 1981, 1984). Given that prehension is planned with the purpose of the action in mind (Grafton, 2010; Flatters et al., 2012; Schubotz et al., 2014), we anticipated changes in both the temporal- and spatial components of reaching and grasping due to changes in material appearance of cups, in which we expected the participants to trade speed for precision when interacting with cups that required cautious manipulation due to their disposition (Fitts, 1954; Battaglia and Schrater, 2007; Petrenel et al., 2017). Besides recording maximum velocity (peak velocity) in reaching and transportation movements, we were especially interested in the adjustment time period in reaching, that is the deceleration period between the peak velocity of the reach and the moment of object contact. For the reason that such temporal phase reflects movement planning based on the object’s identity rather than its location alone (Pryde et al., 1998; Egmose and Køppe, 2018; the term adjustment time is originally used by Rocha et al., 2013). We were also interested in the size of the maximum grip aperture (MGA) during reaching, and how early the MGA appeared for the different types of material properties when reaching for the cups. Moreover, we examined if the same material properties examined for the temporal data, influenced the spatial components of prehension, in particular the selection of grip position during the object contact. Grasping thin-walled deformable objects like paper cups requires a careful movement by the hand and appropriate grip position, where one needs to anticipate not only the weight of the cup but also its local stiffness points and its COM. Such anticipation is important, because applying too much grip force when grabbing a cup will dent it and cause a spillage of its content, while applying too little grip force will result in rotation and failing to hold the cup. Moreover, pouring a liquid into the cup alters its COM, and increases its density, which consequently creates a harder substance. We therefore expect grip preparation and anticipated hardness to be reflected in both the collected temporal- and the spatial data.

To summarize, we performed a seated reaching task to examine the contribution of weight of a material and surface gloss in reaching, grasping, and transporting paper cups. We were especially interested in the spatio-temporal data of the first encounters with the objects during the experiment, as they reflect expectations based visual properties and general knowledge of materials, rather than the direct tactile information. Specifically, we were interested in knowing if the motor movements and selection of grip position involved visual perception of hardness. That is, if a relationship exists between perceived glossiness and anticipated hardness. If the spatio-temporal components of the hand movements are affected by the plausible relationship between surface gloss and rated hardness, it would indicate a strategy, in which an association between what a material looks like and what it feels like, is anticipated in order to facilitate prehension. On the other hand, if no such relationship is found, it would indicate that the two material properties are processed independently.

## Materials and Methods

### Participants

Twenty right-handed participants (seven females) with average age of 28 ± 6 years were recruited on Lund University campus to participate in the study. All participants gave their consent to participate in the study. Due to technical error when collecting the data, few of the participants had missing data in several trials because the cameras could not detect the reflective markers due to their small size and proximity to one another. Six participants were therefore removed from the analysis, and instead we report an analysis for 14 right-handed participants (five females) with the average age of 29 ± 8 years. Considering the smaller sample size than originally planned, *a posteriori* sensitivity power analysis was conducted to determine the minimum detectible effect size for our sample size in terms of Cohen’s f, using the power analysis software G^∗^Power 3 (Faul et al., 2007). The power analysis for our sample size (*N* = 14) allowed us to identify a medium effect size of 0.27 Cohen’s f, with 80% power, and alpha of 0.05.

### Stimuli

**FIGURE 1:**
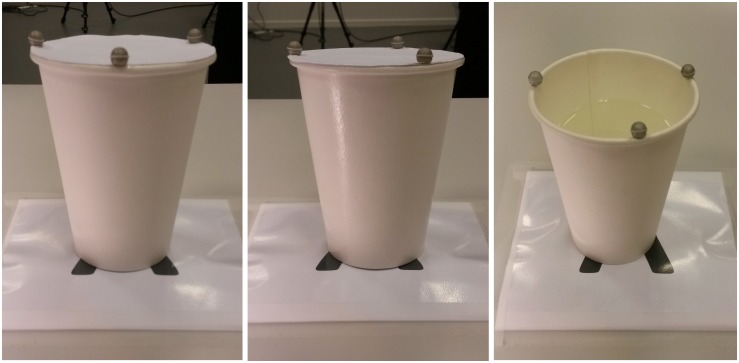
Examples of the experimental stimuli: matte stimulus with hidden content **(left)**, shiny stimulus with hidden content **(center)**, and matte stimulus with visible content **(right)**.

**FIGURE 2:**
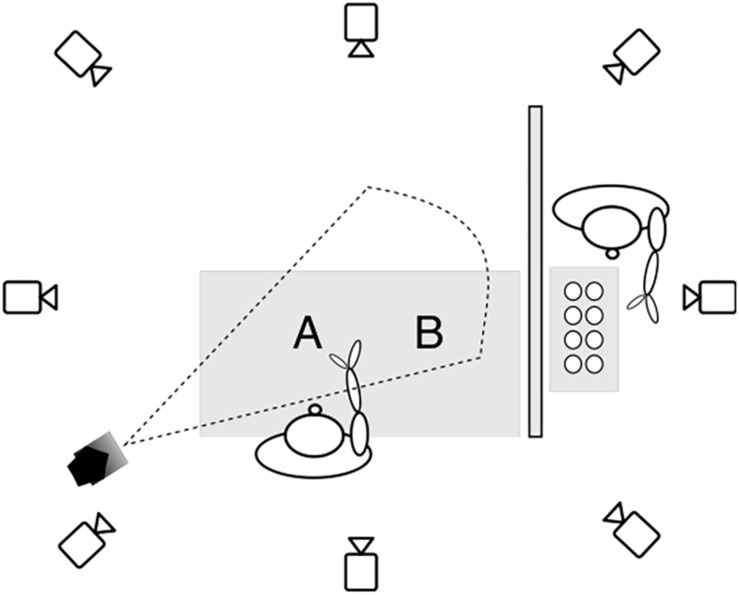
The experimental setup in the motion capture studio. The cups were kept hidden behind a divider and presented by the experimenter one at a time to the participant on location A. Then the participant reached for the cup on location A and transported it to location B. The horizontal distance between locations A and B is 40 cm, and the vertical distance between location A and the start position is 40 cm. The spotlight is positioned on the left of the participant with a distance of 140 cm from location A, casting light diagonally.

The experimental stimuli were eight drinking cups made of white paper lined with wax. The cups were presented either in their original appearances, matte (*N* = 4) or were altered to have shiny appearances (*N* = 4), by first applying two layers of glossy varnish using a soft foam roller to achieve smooth texture, then a permanent clear lacquer spray with high-gloss finish. Examples of the stimuli are seen in Figure 1. Source Four^®^ jr^TM^ ellipsoidal reflector spotlight with 575W high performance lamp with 50° beam angle (Electronic Theater Controls, Inc.) was positioned on the left of the participant, casting the light diagonally to make the shiny appearance of the stimuli more apparent (see Figure 2). The distance between the spotlight and the stimulus when located on position A was 140 and 180 cm when on position B. The stimuli had the shape of a conical frustum and had the capacity for 35 cl, and measured 114 mm in height and 57 mm outer diameter at the bottom of the cup and 87 mm at the top, and weighed 11 g. Each appearance, matte and shiny, had two empty cups and two cups with added weight (liquid), in which one of each had a lid on, a white printing paper, to hide the content of the cup. The added weight was a liquid at room temperature, and weighed together with the cup 252 g. With emphasis on our test exemplar, and according to Alt et al. (2015) model of grasp planning for deformable objects, there are three possible surface areas that afford secure object contact when lifting deformable cups, namely the base and the top due to their attached supportive rings, and the cup’s COM. Holding the cups close to the COM is desirable as it prevents object rotation, and ensures a surface space above it during an upward lifting for repositioning the hand in case the cup starts to slide downward due to its weight. We asked our participants to grab the cups as they would normally do when reaching for a cup, that is to position their grip somewhere on the surface between the base and the top of the cup, using their whole hand.

### Motion Capture System

The Qualisys motion capture system (Qualisys AB, Gothenburg, Sweden) operated with eight high-speed infrared cameras (Oqus 5 + series) was used to capture the motion data in three dimensions. The infrared cameras together with Qualisys track manager (QTM), a tracking software, recorded and located the position of each reflective marker in a three-dimensional space and in real-time. The motion capture system was also operated with one video camera (Oqus 210c), which was used for post-processing. The infrared cameras recorded at the sampling rate of 100 Hz. Five spherical markers made of reflective polystyrene (7 mm in diameter) were attached on the fingernails of each finger, and three markers were attached on the top of each experimental stimulus (*N* = 8). Marker positions were tracked in three dimensions, and coordinate values X (front–back), Y (left–right), Z (up–down), were exported into MATLAB 8.1.0.604 and R (R2013a) for further evaluation. The motion capture system was calibrated for each participant using a Wand calibration system, which consists of two calibration objects. A stationary L-shaped reference object (200 mm × 350 mm at size), which indicated the orientation and origin of the coordinate system (X-, Y-, and Z-axes), and a T-shaped wand (600 mm distance between the two reflective markers on the horizontal line), which was moved through the volume defining the experimental space. Calibration with average tracking residuals per camera below 0.4 mm was considered suitable for the experiment.

### Design and Procedures

Each participant was given chance to get familiar with the experimental task, in which they went through one hypothetical trial of reaching and transporting an object from location A to location B. Next, each participant went through five blocks of test trials, where each block comprised of eight trials, in which each stimulus was presented once, a total of 40 trials per subject. All trials were randomized and counterbalanced across participants. The eight stimuli were four matte and four shiny cups, in which half of them contained liquid and the other half contained nothing. Then, half of the cups from each surface appearance were presented with a lid on to hide their content, and the other half with visible content (no lid). A seven-points semantic differential scale was used to collect subjective ratings of the stimuli in the study, where the participants verbally rated the visual appearance of the eight stimuli using the three bipolar dimensions; heaviness, hardness, and glossiness, both before and after interacting with the stimuli. The scales were as follow. *Heaviness:* if you were to lift the object how heavy would it feel? Low values represent light (light as a feather) and high values represent heavy (heavy as a book), ranging from (1) Very light, (2) Light, (3) Somewhat light, (4) Neutral, (5) Somewhat heavy, (6) Heavy, (7) Very heavy. *Hardness:* if you were to touch the object, would you be able to change the shape of the object using your hand? Low values represent soft (easily with a small force) and high values represent hard (difficult with a small force), ranging from (1) Very soft, (2) Soft, (3) Somewhat soft, (4) Neutral, (5) Somewhat hard, (6) Hard, (7) Very hard. *Glossiness: how shiny is the object?* Low values represent matte (matte as a writing paper) and high values represent shiny (shiny as a mobile phone screen), ranging from (1) Very matte, (2) Matte, (3) Somewhat matte, (4) Neutral, (5) Somewhat shiny, (6) Shiny, (7) Very shiny. The feather used as a reference was a rook feather that weighed less than a gram that are usually found around the university campus, and the book weighed 406 g (height 210 mm, length 150 mm, and width 13 mm).

The total runtime was approximately 45 min, and the procedures were the following. The participant sat in front of a table with his/her eyes closed, left hand rested under the table and right hand rested on a marked start/end position on the table. The participant was told to always keep their hand on that position at the beginning and at the end of each trial. Each experimental trial started after the experimenter placed a new stimulus on location A, taken from a pool of stimuli hidden behind a cardboard, and signaled the participant to open the eyes and start. During the experiment, the stimuli were placed on thin soft coasters to avoid any loud sound feedback when contacting the experimental table, which could give away the weight of the cup. Then, the participant reached for the stimulus on location A and moved it to location B on the right, then released the hand and returned the hand back to start/end position. The participants were allowed to approach the cups using the whole hand (all fingers), to reach for the cups and transport them as quickly and accurately they could, and were asked to position the grip somewhere between the base and the top of the cup during the object lifting. Before the first block of trials, the participants were asked to verbally rate the experimental stimuli and again in the final block after they transported them. When the participant had completed the experiment, he/she was debriefed and thanked.

### Data Analysis

The data was analyzed using the statistical software R (R Core Team, 2018) and MATLAB 8.1.0.604 using the MoCap Toolbox (Burger and Toiviainen, 2013). Linear mixed effect models (LMMs) were fitted for the spatio-temporal data using the *lme4* package in R (Bates et al., 2015). All models were assigned a random intercept for each participant to account for individual differences. Deviations from normality were determined by visual inspection using Q–Q probability plots. The results were analyzed in the following manner.

First, the motion capture recordings were coded for the eight stimuli and for each finger using the Qualisys software. For current study we were only interested in the position of the grip center on the stimuli rather than the spread of the hand when grasping the cups, thus we only included the recordings for three fingers, thumb, index- and middle finger for our analysis. Then, the Euclidean norms were used to calculate the magnitudes of velocities for the two selected motor sequences, reaching and transporting, using the MoCap Toolbox in Matlab. This was done to create one informative vector based on the length of the three-dimensional velocity vector, instead of working with three vectors, one for each direction (X, Y, Z). Afterward, the data was exported to R for further statistical analysis. The relevant motor sequences, reaching and transporting, were then visually identified and coded while the remaining ones were discarded. An onset of a reaching movement was determined when the participant moved the hand at the velocity speed above 30 mm/s over a minimum of 10 successive samples, from the starting position to reach for the stimulus in location A, and the grip (object contact) was determined when the velocity speed returned to below 30 mm/s over a minimum of 10 successive samples. The transportation movement consisted of lifting the stimulus from location A and transporting it to location B, using the same onset and offset time thresholds as used for the reaching movements. Adjustment time in reaching was measured as the duration of the temporal span between the peak velocity in reaching and the end of the reaching sequence, measured in milliseconds and peak velocity was defined as the maximum speed measured in velocity (mm/s) for each of the motor sequences: reaching and transporting. The temporal variables, adjustment time in reaching, peak velocity in reaching, and peak velocity in transporting, were analyzed using repeated measures ANOVAs and linear mixed model regression analysis (LMM).

Second, a spatial analysis was performed to examine the effect of material properties on the MGA and the selection of grip position. The COM of the cups was calculated for the two different weights, empty- and filled cups, by calculating the geometric centroid for a conical frustum (formula 1) and COM that included the object weight (formula 2), as seen in the following formulas.

(1) $$C\,O\,M=\frac{h\,\left(R_{1}^{2}+2\,R_{1}\,R_{2}+3\,R_{2}^{2}\right)}{4\,\left(R_{1}^{2}+R_{1}\,R_{2}+R_{2}^{2}\right)}$$

(2) $$C\,O\,M\,w\,e\,i\,g\,h\,t=\frac{L_{w}\,C\,O\,M+C_{w}\,C\,O\,M}{L_{w}+C_{w}}\,$$

In formula 1, the centroid for the geometric shape of the cups and the liquid along the z-axis is calculated separately, in which *h* stands for respective vertical height of either the cup or the liquid in mm. *R*_1_ represents the initial radius in mm for the smaller surface area at the base of the cup and the liquid, whereas *R*_2_ represents the final radius in mm for the larger surface area at the top of the cup and the liquid. In formula 2, weight of the cup and the liquid are incorporated to get the COM for empty and filled cups, based on both the geometric shape and the weight of the cups and the liquid. Here COM represents the geometric centroid for the cups and the liquid respectively, *L*_*w*_ stands for the weight of the liquid in grams, and *C*_*w*_ for the weight of the cups in grams. An empty cup had its COM, measured along the vertical axis from the initial radius at the base of the cup to the final radius at the top, at 65 mm, whereas a cup with liquid had its COM at 46 mm. Next, we calculated the maximum distance (mm) between the thumb and the index finger during the reaching movement before the object contact to get the MGA, in which we were interested in both the size of the MGA for the different types of material properties and its timing. After, we examined the position coordinates of the grip during the object contact. First, the vertical position of the grip center (z-dimension) based on the average position coordinates of the thumb, the index finger, and the middle finger was found. Second, the degree of grip deviation from the COM, measured as the grip center’s vertical distance from each object’s COM in mm, in which positive values represented position coordinates above the COM and negative values represented position coordinates below the COM. The size and the position of MGA were analyzed using repeated measures ANOVA, whereas the selection of grip position, and data from the first block of trials, were analyzed using linear mixed effects regression models.

Finally, the relationship between the object properties (surface and content) and the rated properties, heaviness, hardness, and glossiness, both as pre-reaching ratings and post-transportation ratings, were examined using cumulative link mixed models (CLMMs) using the *ordinal* package in R (Christensen, 2018). Separate regression models were then conducted to examine the spatio-temporal data from the first block of trials, to see if the expected properties based on the pre-reaching ratings were reflected in the motor movements of the hand, or in the selection of grip position.

## Results

We expected distinct temporal- and spatial patterns in motor movements, MGA, and grip position for the different types of cups due to learned associations between material appearance and intrinsic properties. Furthermore, we wanted to explore if the motor movements, MGA, as well as the position of the hand on the cups, were guided by the expected properties, especially visually perceived glossiness and hardness.

### The First Impression of the Material Properties

**FIGURE 3:**
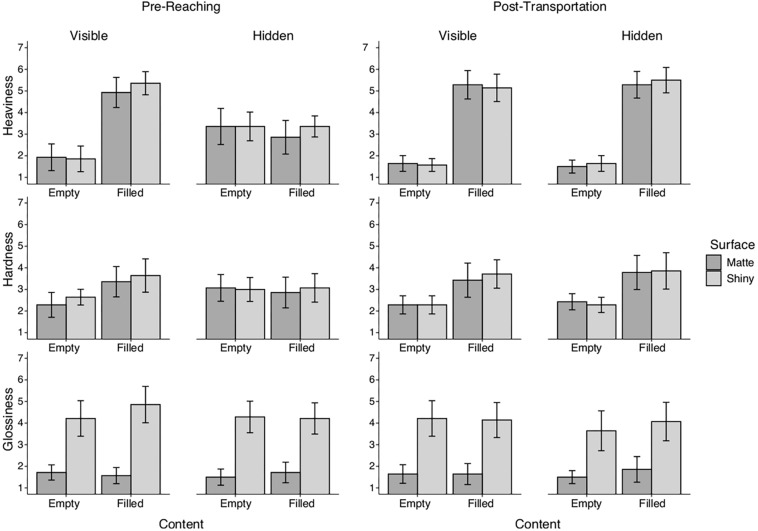
Mean rated heaviness, hardness, and glossiness for the eight stimuli. **(Left)** Ratings collected before reaching for the stimuli. **(Right)** Ratings collected after transporting the stimuli. Error bars are 95% confidence intervals. The two graphs suggest that the pre-reaching ratings are based on expectations whereas the post-transportation ratings are based on sensorimotor information. The figure also suggests strong categorical impressions of the properties, heaviness, and glossiness (bimodal values) whereas the impression of hardness is less defined.

We were interested to know in what way the object properties influenced the subjective ratings, specifically before the first interaction with the cups. Thus, a cumulative link mixed regression analysis (CLMM) was conducted to examine the relationship between the object properties (surface appearance and content) and the rated properties (heaviness, hardness, and glossiness) based on the pre-reaching ratings. The expected properties represented as rated heaviness, hardness, and glossiness were computed by averaging over the seven items of the scale for each of the stimuli, separately for the pre-reaching ratings and the post-transportation ratings. The mean ratings of the scales for the eight stimuli are shown in Figure 3.

The CLMM analysis revealed statistically significant relations between the physical- and the rated properties. We found *rated heaviness* to be largely affected by the content of the cups, that is whether the cups contained liquid or not, *b* = 1.98, *SE* = 0.12, χ^2^(1) = 299.94, *p* < 0.001. According to the pre-reaching ratings, the participants rated the filled cups to be heavier (*M* = 5.14, *SD* = 1.08) than the empty cups when the content was visible to them (*M* = 1.89, *SD* = 1.03), whereas rated heaviness was similar for the two object weights when the content of the cup was hidden using a lid (filled: *M* = 3.11, *SD* = 1.13; empty: *M* = 3.36, *SD* = 1.28). We also found the surface appearance, that is whether the cups had matte or varnished appearance, influenced the rated heaviness, *b* = 0.36, *SE* = 0.11, χ^2^(1) = 11.22, *p* < 0.001, in which the shiny cups (*M* = 3.61, *SD* = 2.02) got rated heavier than matte cups (*M* = 3.42, *SD* = 1.89), both when the content was visible and hidden (shiny: *M* = 3.36, *SD* = 0.99; matte: *M* = 3.10, *SD* = 1.40). Intriguingly, we found *rated hardness* to be influenced by both the object weight and the surface appearance, in which filled cups got rated harder (*M* = 3.50, *SD* = 1.26) than empty cups when the content of the cups was visible (*M* = 2.46, *SD* = 0.84), *b* = 1.41, *SE* = 0.14, χ^2^(1) = 115.86, *p* < 0.001, whereas when the content was hidden the filled and empty cups were rated similar in terms of hardness (filled: *M* = 2.96, *SD* = 1.17; empty: *M* = 3.04, *SD* = 1.00). Looking into the role of surface appearance in rated hardness we found when the content was visible, the participants rated the shiny cups (*M* = 3.14, *SD* = 1.15) to be harder than the matte cups (*M* = 2.82, *SD* = 1.22), *b* = 0.63, *SE* = 0.13, χ^2^(1) = 24.78, *p* < 0.001. Whereas, the participants rated the two types of appearances to be similar in hardness when content was hidden (shiny: *M* = 3.04, *SD* = 1.04; matte: *M* = 2.96, *SD* = 1.14). For *rated glossiness* we found surface appearance largely influenced the impression of surface gloss, *b* = 6.89, *SE* = 0.28, χ^2^(1) = 1421.9, *p* < 0.001, in which shiny cups got rated shinier than matte cups, both when the content was visible (shiny: *M* = 4.54, *SD* = 1.45; matte: *M* = 1.64, *SD* = 0.62), and when the content was hidden (shiny: *M* = 4.25, *SD* = 1.24; matte: *M* = 1.61, *SD* = 0.74). No significant effect of object weight (i.e., content) was found on rated glossiness, *b* = 0.13, *SE* = 0.11, χ^2^(1) = 0.23, p = n.s.

### Velocity Speed and Adjustment Time in Reaching

Overall, the three experimental conditions, (1) the content of the cups (Content: Empty vs. Filled), (2) the appearance of the surface (Surface: Matte vs. Shiny), and (3) the visibility of the content (Feedback: Visible vs. Hidden) had no effect on the peak velocity (mm/s) in reaching. A repeated measures within-subjects ANOVA revealed no significant effect for content, surface appearance, or feedback on peak velocity in reaching, over the five blocks of trials: *content* (Empty: *M* = 1039.89, *SD* = 267.26, Filled: *M* = 1011.67, *SD* = 236.26), *F*(1,13) = 2.30, *p* = 0.15.; *surface appearance* (Matte: *M* = 1022.33, *SD* = 268.59, Shiny: *M* = 1029.22, *SD* = 235.54), *F*(1,13) = 0.52, *p* = 0.49.; and *feedback* (Visible: *M* = 1018.38, *SD* = 249.12, Hidden: *M* = 1033.17, *SD* = 255.89), *F*(1,13) = 0.61, *p* = 0.45. All two-way and three-way interactions were non-significant as well (all *p*s = n.s.).

Looking into the planning phase when the participants approached the cups, a repeated measures within-subjects ANOVA with content, surface appearance, and feedback as predictive variables and adjustment time (ms) in reaching as the dependent variable, revealed significant effect for content on the adjustment time over all five blocks of trials, *F*(1,13) = 71.76, *p* < 0.001, η^2^G = 0.10. Overall the participants had longer adjustment times when reaching for filled cups (*M* = 1024 ms, *SD* = 212 ms), compared to empty cups (*M* = 881 ms, *SD* = 139 ms). No other main effect or interaction effects were found for the five blocks of trials together (all *p*s = n.s.). A Kruskal–Wallis test revealed the adjustment time distributions to be significantly non-identical across the five blocks of trials, χ^2^(4) = 14.54, *p* < 0.001, which opted for a further examination of the adjustment times, block by block.

### Comparison of Adjustment Times in Reaching

To examine the role of expectations of material properties in reaching, we conducted a linear mixed model regression analysis on the adjustment times for each of the five blocks of trials, with particular interest in the first and the last block of trials for comparison purposes. Each model consisted of three predictive variables as fixed factors, which were the object weight (content), surface appearance, and the visibility of the content (feedback), and a random intercept for each participant. The LMM analysis for the first block of trials revealed significant effect of content, in which adjustment time increased when reaching for filled cups (*M* = 1192.32, *SD* = 421.50 ms) compared to empty cups (*M* = 939.46 ms, *SD* = 299.88 ms), *b* = 252.86, *SE* = 52.08, χ^2^(1) = 23.68, *p* < 0.001. The analysis also showed the adjustment time to significantly increase when the participants reached for the cups with the matte appearance (*M* = 1120.54 ms, *SD* = 415.02 ms), compared to when the participants reached for the cups with the applied varnish (*M* = 1011.25 ms, *SD* = 349.03 ms), *b* = 109.29, *SE* = 52.08, χ^2^(1) = 4.40, *p* < 0.05. The visibility of the content had, however, no effect on the deceleration phase in reaching, as no significant main effect was found for the variable feedback on adjustment time for the first block of trials, *b* = 70.71, *SE* = 52.08, χ^2^(1) = 1.84, p = n.s.

For comparison purposes, we conducted a LMM analysis for the last block of trials (block 5), which revealed both content and surface appearance to have significant effect on the adjustment time in reaching, although the effect of surface appearance on the adjustment time in the last block of trials was much smaller compared to the adjustment time in the first block of trials. The adjustment time increased when reaching for the filled cups (*M* = 1006.79 ms, *SD* = 287.53 ms), compared to the empty cups (*M* = 850.36 ms, *SD* = 237.95 ms), *b* = 156.43, *SE* = 38.75, χ^2^(1) = 16.30, *p* < 0.001, and increased when reaching for the cups with the matte appearance (*M* = 967.14 ms, *SD* = 273.17 ms), compared to cups with the varnished appearance (*M* = 890.00 ms, *SD* = 272.21 ms), *b* = 77.14, *SE* = 38.75, χ^2^(1) = 3.96, *p* < 0.05.

**FIGURE 4:**
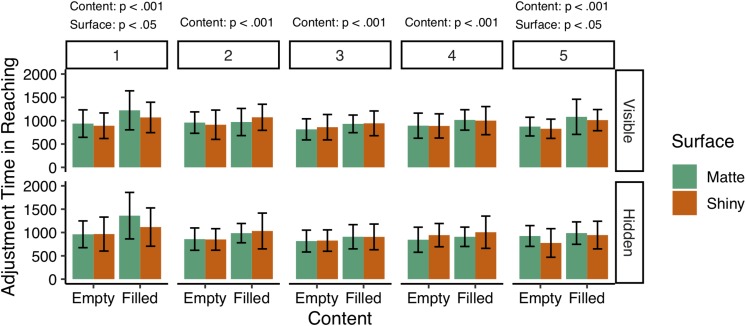
Average adjustment times (ms) in reaching for each block of trials (*N* = 5) based on the cups content and surface appearance (material). Both variables, the weight of the cups and the surface gloss, had significant effect on the adjustment times during the first block of trials, and the last block of trials. The participants adopted longer adjustment times when reaching for cups with either matte appearance, or were filled with liquid (heavier). No significant effect was found for the feedback condition, indicating no difference in adjustment times when reaching for cups with or without lid.

For the remaining blocks (2, 3, and 4), the LMM regression analysis revealed no significant effect of surface appearance (matte vs. shiny) or feedback (visible vs. hidden content) on the adjustment times in reaching (all ps = n.s.). Only the content of the cups, that is the object weight, influenced the adjustment time for those blocks of trials. For the second block of trials, the filled cups had significantly longer adjustment times (*M* = 1015.71 ms, *SD* = 291.67 ms) compared to empty cups (*M* = 895.36 ms, *SD* = 252.63 ms), *b* = 120.36, *SE* = 32.66, χ^2^(1) = 13.58, *p* < 0.001. Similarly, filled cups had longer adjustment times (*M* = 922.50 ms, *SD* = 243.22 ms) compared to empty cups (*M* = 824.69 ms, *SD* = 235.12), *b* = 92.86, *SE* = 21.84, χ^2^(1) = 18.08, *p* < 0.001 in the third block of trials. As well as in the fourth block of trials, in which filled cups (*M* = 982.86 ms, *SD* = 270.54 ms) had longer adjustment time compared to empty cups (*M* = 892.50 ms, *SD* = 256.81), *b* = 90.36, *SE* = 28.46, χ^2^(1) = 10.08, *p* < 0.001. The results are illustrated in Figure 4.

In order to examine if the varnished surface appearance had enhanced role in motor planning when the participants could not estimate the weight of the cups based on their content (cups with lids). We further analyzed the reaching movement for cups with hidden content only. Using LMM regression analysis on adjustment times when reaching for cups with lids on, we found no difference in adjustment times for the two types of surface appearances (matte vs. shiny). Both when examining all blocks of trials together (Matte: *M* = 955.64 ms, *SD* = 180.06 ms; Shiny: *M* = 936.86 ms, *SD* = 188.06 ms), *b* = −18.79, *SE* = 33.18, χ^2^(1) = 0.32, p = n.s., and when examining only the first block of trials (Matte: *M* = 1161.07 ms, *SD* = 447.88 ms; Shiny: *M* = 1041.43 ms, *SD* = 388.30ms), *b* = −22.21, *SE* = 31.84, χ^2^(1) = 0.49, p = n.s. In fact, we found no significant effect of visibility (feedback: visible vs. hidden) on the adjustment times in reaching, neither for all blocks of trials together, *b* = −13.00, *SE* = 22.96, χ^2^(1) = 0.32, p = n.s., nor for the first block of trials, *b* = 70.71, *SE* = 59.05, χ^2^(1) = 1.43, p = n.s.

### Grip Aperture and Grip Position

Before we examined the selection of grip position for the different types of material properties, we looked into the grip preparation moment in reaching, in which we examined the size of the MGA and the timing of it. First, we measured the MGA before object contact, defined as the maximum metric distance (mm) between the thumb and the index finger, for each stimulus per participant. Using repeated measures ANOVA, we found no significant effect of content, surface appearance, or feedback, on the size of the MGA when analyzing all blocks of trials (all *p*s = n.s.). Moreover, LMM regression analysis for the first block of trials revealed no significant effect of the three predictive variables on MGA size either (all *p*s = n.s.). Overall, the average size of MGA was 119.61 mm (*SD* = 12.98 mm). Next, we looked into the timing of the MGA as measured from the movement start to the moment the aperture reached its maximum value. Using repeated measures ANOVA on all blocks of trials, we found the absolute timing of the MGA was not guided by the object properties or their visibility. Furthermore, LMM regression analysis on the first block of trials revealed no significant effect for the same variables on the absolute timing of MGA (all *p*s = n.s.). On the average, the MGA for the different types of cups was at 945.86 ms (*SD* = 296.09 ms) after the movement start.

**FIGURE 5:**
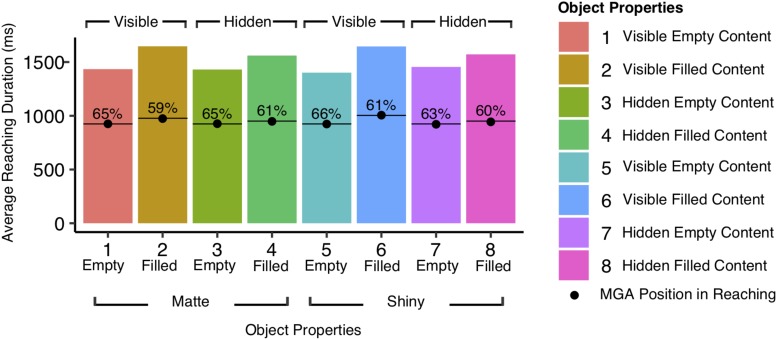
Average duration times in reaching (ms) for the different types of cups presented with respective average timing of the maximum grip aperture (MGA). The figure demonstrates MGA to occur earlier in time when reaching for cups with filled content (*M* = 61.67% after movement start) compared to empty and consequently lighter cups (*M* = 66.78% after movement start). This effect was present when the timing of MGA was examined in relation to the reaching duration times, but was absent when the timing of MGA was examined as the absolute time from movement start.

We found, however, variations in the time point of MGA in proportion to the duration time of reaching per condition. As seen in Figure 5, the duration times of reaching differs for the different types of cups depending on their properties, with average duration time ranging from 1401.14 ms (*SD* = 528.67 ms) to 1646.43 ms (*SD* = 699.55 ms). The figure also shows that the timing of MGA varies in proportion to the different presented duration times. Further examination using a repeated measures ANOVA revealed significant effect of object weight (content: filled vs. empty) on the time position of MGA in relations to the reaching duration time for the different types of cups. The MGA occurred earlier in time when reaching for filled cups (*M* = 61.67%, *SD* = 6.32%) compared to empty cups (*M* = 66.78%, *SD* = 7.53%), *F*(1,103) = 14.57, *p* < 0.001. A similar ANOVA analysis on the first block of trials revealed object weight had a significant effect on the timing of MGA, as we found MGA occurred earlier when the participants reached for filled cups (*M* = 58.14%, *SD* = 9.07%) compared to empty cups (*M* = 65.86%, *SD* = 10.52%), *F*(1,103) = 16.71, *p* < 0.001. No other main effects or interactions were found significant (all *p*s = n.s.).

**FIGURE 6:**
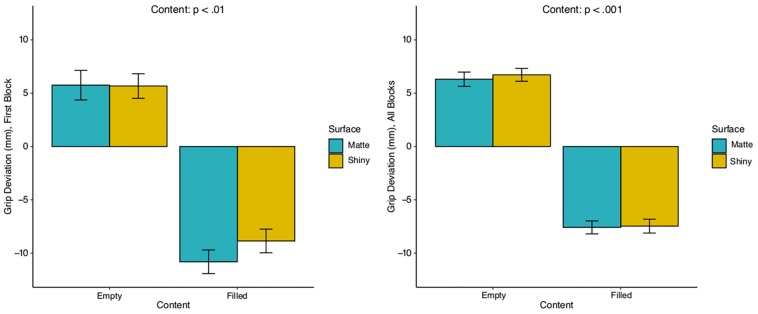
Grip deviations from the center of mass (COM) in mm as a function of manipulated properties, for the first block of trials **(left)**, and across all blocks of trials **(right)**. The COM is measured from the base of the cups and is positioned at 65 mm for empty cups, and 46 mm for filled cups and is represented respectively as a zero on the y-scale. The error bars are standard errors. Significant difference was found between empty and filled cups. Surface is shown for comparison purposes.

The selection of grip position was examined using the vertical spatial position of the grip center of the hand, calculated as the average horizontal distance between the thumb, index-, and middle finger, using LMM. We were both interested in the grip position measured as the distance from the base of the cup in mm, and if the position deviated from the cups’ center of the mass. When examining all blocks of trials together, the comparison of a full model to a nested model, revealed that the removal of *content* (empty, filled), χ^2^(2) = 66.58, *p* < 0.001, as main effect significantly reduced the model fit, whereas the removal of *surface* (matte, shiny) did not significantly affect the model fit, χ^2^(2) = 0.19, p = n.s. Further regression analysis on the selection of grip position using a model with only content as fixed effect revealed grip position to be guided by the content of the cups, in which filled cups were grasped lower than empty cups, *b* = −4.11, *SE* = 0.49, χ^2^(1) = 70.73, *p* < 0.001. Although the cups were on the average grasped at their respective COM, a further examination on the direction of grip deviations from the two types of COM, revealed an upward grip deviation for the empty cups, whereas we found a downward deviation for the filled cups with heavy properties, *b* = −13.90, *SE* = 0.68, χ^2^(2) = 516.5, *p* < 0.001. Moreover, a LMM analysis on the selection of grip position for the first block of trials, using a model with only content as the fixed effect, revealed grip position to be affected by the content of the cups, in which filled cups were grasped significantly lower than empty cups, *b* = −2.26, *SE* = 0.81, χ^2^(1) = 7.72, *p* < 0.01. Figure 6 shows the relationship between the grip position and the COM of the cups, in which we found an upward grip deviation from COM for empty cups and downward deviation for filled cups, *b* = −16.57, *SE* = 1.10, χ^2^(2) = 160.11, *p* < 0.001.

### Comparison of Transportation Velocities

Overall, a repeated measures ANOVA revealed the participants adopted slower transportation speed when reaching for filled cups (*M* = 657.46 mm/s, *SD* = 112.68 mm/s), compared to empty cups (*M* = 834.01 mm/s, *SD* = 171.62 mm/s), *F*(1,13) = 73.77, *p* < 0.001, η^2^G = 0.36. No other main effect or interaction effects were found based on the overall performance (all *p*s = n.s.). A Kruskal–Wallis test revealed transportation peak velocity distributions to be significantly non-identical across the five blocks of trials, χ^2^(4) = 16.31, *p* < 0.001, which called for further examination.

**FIGURE 7:**
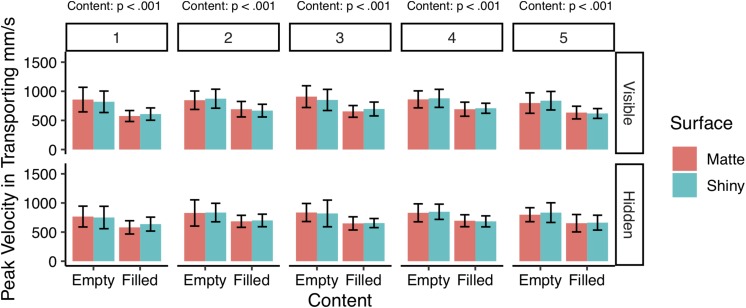
Peak velocity (mm/s) and standard errors when transporting the cups from location A to location B. The graphs show that the filled cups are moved significantly slower than empty cups (all ps < 0.001), whereas no significant effect was found for surface appearance or visibility of the content on peak velocity in transporting.

Figure 7 shows the comparison between the five blocks of trials for the velocity measurements in transportation, in which an LMM regression analysis revealed a dominant effect of object weight (content) on the transportation speed when moving the cups. For the first block of trials, the transportation speed (peak velocity) was significantly slower for filled cups (*M* = 600.48 ms, *SD* = 108.65 ms) compared to empty cups (*M* = 798.52 ms, *SD* = 191.55 ms), *b* = −198.03, *SE* = 18.30, χ^2^(1) = 117.00, *p* < 0.01. As well as for block 2 (Filled: *M* = 686.18 ms, *SD* = 111.36 ms; Empty: *M* = 845.79 ms, *SD* = 686.18 ms), *b* = −159.61, *SE* = 18.67, χ^2^(1) = 73.12, *p* < 0.01. For block 3 (Filled: *M* = 663.76 ms, *SD* = 102.77 ms; Empty: *M* = 853.93 ms, *SD* = 187.68 ms), *b* = −190.16, *SE* = 15.73, χ^2^(1) = 146.10, *p* < 0.01. For block 4 (Filled: *M* = 694.92 ms, *SD* = 99.44 ms; Empty: *M* = 854.77 ms, *SD* = 143.87 ms), *b* = −159.85, *SE* = 12.19, χ^2^(1) = 172.00, *p* < 0.01. For block 5 (Filled: *M* = 641.95 ms, *SD* = 117.86 ms; Empty: *M* = 817.06 ms, *SD* = 154.19 ms), *b* = −175.11, *SE* = 16.91, χ^2^(1) = 107.23, *p* < 0.01. No significant effect was found for surface appearance (material) or for the visibility of the content (feedback), all *p*s = n.s.

### Expected Material Properties in Prehension and Transportation

**FIGURE 8:**
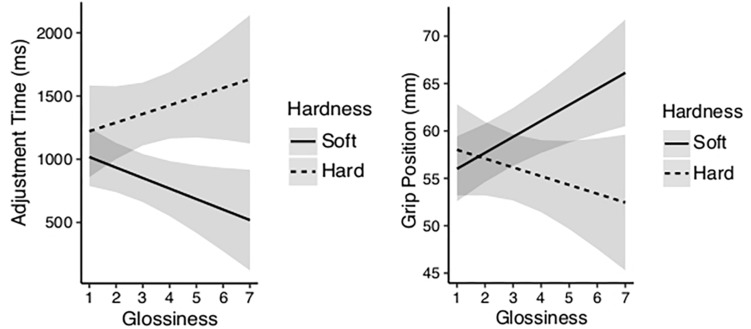
**(left)** For the first block of trials, the estimated adjustment time based on LMM analysis for the interaction between the properties glossiness and hardness as based on the pre-reaching ratings. The figure shows cups assigned hard properties to have longer adjustment time than cups assigned soft properties, and that distinction to increase the shinier the cups got rated. **(right)** Estimated grip position based on LMM analysis for the same rated properties. The figure shows grip position on rated soft cups to move upwards in vertical direction the shinier they got rated, and move downwards for rated hard cups the shinier they got rated.

Figure 8 reveals the relationship between the rated properties and the spatio-temporal data. A LMM regression analysis on the effect of pre-reaching ratings on adjustment times for the first block of trials, revealed reaching to be significantly affected by the expectation of glossiness and hardness. We found rated glossiness, *b* = −144.58, *SE* = 60.51, χ^2^(1) = 4.23, *p* < 0.05, and the interaction between rated hardness and glossiness, *b* = 39.43, *SE* = 19.36, χ^2^(1) = 4.14, *p* < 0.05, to influence the adjustment time, whereas rated heaviness was found to have no effect on the adjustment time in reaching for the first block of trials, *b* = 105.68, *SE* = 55.93, χ^2^(1) = 3.48, *p* = 0.06. The figure shows longer planning phase, characterized by the longer adjustment times in reaching for cups with hard properties, which got longer the shinier the cups got rated. In comparison, the planning phase in reaching was shorter for cups with rated soft properties, and it got shorter the shinier the cups got rated. Indicating a distinct categorical perception for the two types of relationship directions. Moreover, when examining the relationship between the pre-reaching ratings and the selection of grip position, in which we found grip position to be affected by the rated glossiness, *b* = 2.36, *SE* = 0.83, χ^2^(1) = 7.77, *p* < 0.01, and by the interaction between glossiness and hardness, *b* = −0.58, *SE* = 0.27, χ^2^(1) = 4.59, *p* < 0.05, indicating that the spatio-temporal components of reaching and grasping are to some extend based on the visual impression of the material properties.

When examining the peak velocity for transporting the cups, no effect was found for the rated glossiness and hardness, neither when examining the pre-reaching ratings nor the post-transportation ratings (all ps = n.s.). A significant effect, however, was found for perceived heaviness based on the post-transportation ratings collected after the last block of trials, χ^2^(1) = 14.49, *p* < 0.001. The results suggest that the expected properties as based on the material appearance of the cups has a role in guiding reaching and grasping for the first encounters, but after the object lifting and during the transportation of the cup, the sensorimotor information based on the object weight becomes known, and consequently next physical interactions with similar objects are characterized by that updated knowledge.

## Discussion

Visual perception of material properties has largely been studied via passive viewing of either static or dynamic images of materials (e.g., Sharan et al., 2014). Consequently, less is known about the function of material perception in real environments when interacting with objects made of familiar materials. Here, we examined the role of visual perception of surface gloss and object weight in reach-to-grasp planning when handling familiar objects. Furthermore, we were interested to know if the visual perception of hardness was reflected in the spatio-temporal measurements of prehension. We therefore additionally examined if perceived hardness had a positive relationship with surface gloss, and if such relationship was incorporated in the handling of the objects.

According to the dual visuomotor channel theory in motor planning (Jeannerod, 1981, 1984), prehension is comprised of two movement components, reaching and grasping, encoded by two distinct visuomotor pathways that project from the visual cortex to the motor cortex. Due to the anatomical distinction, the two components are controlled separately, in which reaching is solely guided by the extrinsic properties such as the distance between the hand and the targeted object or the object’s orientation, whereas grasping is based on the object’s intrinsic properties such as its size. However, later studies have revealed that both intrinsic and extrinsic properties of objects can influence the motor control in reaching, thereby expanding the role of intrinsic properties in prehension (e.g., Paulignan et al., 1991a, b).

In our study, we found adjustment times in reaching were influenced by not only the object weight but also by the properties that described the materials the cups were made of, that is whether the cups had varnished appearance or not. In comparison, we found peak velocity in reaching to be unaffected by those same properties. This suggests that the beginning of reaching movements is for guiding the hand to the targeted location, whereas the deceleration period between the peak velocity and the object contact is for grasp preparation and therefore guided by the precision requirements based on the object properties. A meta-analysis based on 39 reach-to-grasp studies supports in fact such a division. Overall, Egmose and Køppe (2018) found extrinsic properties, such as the distance between the hand and the object, commonly guided the acceleration phase at the start of the reach, whereas the deceleration phase at the end of the movement was usually a preparation period for the grasp and therefore either guided by the object properties or by the abstract end-state goal, such as grasp-to-lift versus grasp-to-throw goals. Recent neurophysiological and neuroimaging studies support such earlier involvement of object properties in reaching as well (Grafton, 2010; Touvet et al., 2014; Turella and Lingnau, 2014; Milner, 2017; Freud et al., 2018).

### Spatio-Temporal Evidence for Object Weight and Surface Gloss

We found the different object properties led to changes in reaching and grasping. The temporal components of reaching were initially guided by the material properties based on the visual appearance of the object and the object weight, while the timing of the MGA, and the selection of grip position were guided by the weight of the object alone.

Overall, we found object weight had a significant influence on adjustment time in reaching, both during the first block of trials, and across all blocks of trials, in which our participants took longer time to reach for heavier cups compared to lighter cups. Moreover, we found stimulus weight and rated heaviness based on the post-transportation ratings, influence the peak velocity during the transportation phase, in which filled cups (i.e., heavier) were moved significantly slower than empty (i.e., lighter) cups. In this respect, our results are in line with previous findings demonstrating longer planning phase of movements for objects made of heavy materials (e.g., brass) (Weir et al., 1991; Fleming et al., 2002; Paulun et al., 2014, 2016). The filled cups were heavier than the empty cups because they comprised of an additional material, water, which is heavier than the paper cups alone. The longer adjustment times when reaching for filled cups could therefore be explained by how the object weight was manipulated. Certainly, the precision requirement is greater for the filled cups, since their weight was created by pouring fluid in them. As a consequence, the participants had to anticipate the material properties of the water based on their knowledge of liquids, to prevent spillage when handling the cups. Interestingly, securing the opening of the cups with lids did not affect the adjustment time in reaching, even though such changes reduced the risk of spilling. Moreover, we found no overall effect of whether the cups had a lid on or not on the temporal components of reaching, which highlights the role of object weight in prehension, although other properties of water might contribute as well. Nonetheless, we found reaching movements are guided by the expected precision requirements, in which objects that require greater precision to be handled without errors are approached slower due to their disposition (Fitts, 1954).

The results also revealed a role of surface appearance in prehension. For the first block of trials, we found adjustment time was significantly shorter for cups with applied varnish, indicating a shorter grasp preparation time needed for such material appearance, compared to the longer planning phase, which we found for cups with matte appearance. Similarly, the regression analysis on the temporal data using the pre-reaching ratings as fixed effects revealed shorter adjustment time the shinier the cups got rated. We found, however, surface appearance to have no effect on the velocity speed when transporting the cups. Interestingly, the kinematic analysis revealed significant effect of object weight on adjustment time for all of the five blocks of trials individually. Whereas we only found significant effect of surface appearance (matte vs. shiny) on adjustment time for the first and the last block of trials, blocks 1 and 5, and not for the central blocks of trials, blocks 2, 3, and 4. It is likely that the participants were more aware of the object properties in the first and the last block of trials, compared to the central blocks due to the experimental task bound in them. In experimental blocks 1 and 5 the participants were asked to rate the cups using the provided scales for heaviness, hardness, and glossiness, whereas the central blocks of trials were conducted without any rating sessions. In block 1 the participants first rated the cups before reaching for them and relocating them, while in block 5 the participants knew they were going to rate the cups after they had relocated them. In other words, the instruction to form subjective impressions of the cups’ properties might have enhanced the changes in the kinematic data.

The results demonstrate that object weight does not have a monopoly when it comes to planning and executing reaching movements. Instead visuomotor planning of reaching involves, to some extent, the expectations of material properties based on the surface appearance of the cup, in our case the glossiness of the cups. Then, after repeated object lifting the motor mechanism is updated by the provided sensorimotor information and the subsequent motor movements are adapted according to the object weight provided by the haptic feedback. Stimulus weight was found to have a prevailing effect on reaching movement after repeated trials, and continued to have an effect after the object contact by either reducing or increasing the transportation speed, depending on the object weight.

Further examination on the grip preparation before object contact, revealed MGA in reaching was not affected by the object properties, weight or surface gloss, as we found no significant effects on MGA size for the different types of cups. Instead, we found the duration times in reaching varied for the different types of properties and further examination revealed object weight (content: filled vs. empty) had a significant effect on the timing of the MGA in relations to the duration times for the different types of cups. The participants reached MGA earlier when reaching for filled cups compared to the empty cups, indicating a longer planning phase required after the MGA for those properties. Surprisingly, this effect on the MGA timing was not found when examining the absolute timing of MGA as measured as milliseconds from the movement start, which suggest the object properties have a role in motor planning after the MGA is reached. Similar findings for aperture in reaching have been reported before by Paulun et al. (2016), who found no effect on the size of MGA in reaching for their tested materials (Styrofoam, Wood, Brass, Slippery Brass), and no effect on its timing, measured as the duration time between the onset of reaching and MGA value. Instead, they found the timing of MGA varied in relations to the duration times, in which MGA occurred earlier when approaching objects made of Brass or Brass with slippery properties, compared to Styrofoam.

When examining the spatial components of the selection of grip position during the object contact, we found no effect of surface gloss. Instead, our results showed a clear effect of object weight, in which the participants positioned their grip at the cup’s COM, rather than simply positioning the grip at the mere center of the cups. Supporting previous findings on the role of perceived mass in prehension (Weir et al., 1991; Brouwer et al., 2006; Eastough and Edwards, 2007; see Smeets and Brenner, 1999 for a review). While the results indicated no influence of surface gloss on the position of the grip during the object contact, it indicated that the participants coordinated their grip position according to the object’s perceived material class. The participants grabbed the cups at their assigned COM, based on the inferred weight, in which they anticipated the liquid filled cups to have lower COM than the empty cups, and positioned their grip accordingly. Interestingly, one would not have expected any grip deviations for liquid filled cups, as the cups are heavy and require precision in grip position to ensure stableness and prevent torque. Regardless, we found the participants positioned their grip center either at the axis of the cups’ COM, or they positioned it below the absolute COM when reaching for the filled cups. A similar effect found by Weir et al. (1991) and Paulun et al. (2016) when reaching for heavy objects. Our introspection suggests that the downward grip deviation from the COM is a strategy to secure a space above the grip in case the cups were heavier than expected and started to slip downward. That way the participants had space to reposition the grip if needed.

### Rated Hardness and Glossiness

Hardness has been extensively studied in haptic perception (e.g., Han and Choi, 2010; Baumgartner et al., 2013), however in the context of visual material perception, little is known about hardness anticipation based on visual features when grasping objects made of materials that vary in compliance. In our study we hypothesized that the expected hardness of the cups would be reflected in the spatio-temporal measurements when reaching for the cups. Moreover, we hypothesized that rated hardness would have a positive relationship with the surface appearance of the cups, that is whether the cups had applied varnish or not, which would as well be reflected in the collected data.

In our study it is likely that the hardness ratings represented the expected density of the cups, as the ratings appeared to be influenced by the presence of the liquid in the cups. The filled cups were perceived harder (less deformable) compared to the empty cups, likely due to the liquidized content of the cups. Filling the cups with liquid creates a force against the inside walls of the cups, and results in perceived harder quality compared to cups without content. In terms of surface gloss, we found our participants rated the cups with the applied varnish to have harder quality compared to the ratings assigned to the matte cups. Moreover, CLMM analysis revealed significant relations between not only rated hardness and the content of the cups, but also between rated hardness and the surface gloss, which favors our assumption that there is a positive relationship between glossiness and hardness. When investigating the relationship between rated properties and the temporal data, we found adjustment time in reaching to be affected by the interaction between the expected glossiness and hardness, based on the pre-reaching data, however, the role of glossiness depended on how soft or hard the cups got rated. The participants reached slower for cups with rated hard qualities, compared to cups with rated soft qualities, and that distinction became larger the shinier the cups got rated. In other words, the planning phase in reaching was long for cups with rated hard properties and got longer the shinier the surface got rated, whereas the planning phase was shorter for cups with rated soft properties, and got shorter the shinier the surface got rated, suggesting a distinct categorical perception for hard and soft surfaces with shiny appearance.

Looking into the spatial data, we did not find surface appearance as an object property to have any effect on the selection of grip position. Regardless, we found cups with rated shiny and hard properties to be grabbed lower than cups with rated shiny and soft properties. We argue that cups with rated hard properties are expected to be denser, and due to the prior knowledge that dense materials tend to be heavy and that glossy surfaces are often slippery, the participants approach those cups with more caution. In comparison, cups with rated shiny and soft properties are expected to be lighter and with lower expected torque, which allows the participant to approach the cups with less precision and higher speed.

It is probable that we rely on stored associations based on material appearances to estimate hardness before interacting with an object. Associations that are based on previous experiences of interacting with objects with similar appearances and certain qualities, that are constantly being updated after every object contact. Fleming (2017) among others have noted that the estimation of hardness of objects before touching them is an indistinct task, as any visual information other than the shape deformation itself are ambiguous. As he cleverly clarifies with an example of an object with wooden appearance that is estimated to have hard qualities, but then when that same object is deformed when touched, it is perceived to have soft qualities despite the appearance usually associated with hardness. Clearly, the shape alteration of the object is a much stronger cue for hardness than the visual surface texture of the object, but some textured information led us to think that the object had hard quality to begin with.

For future research it would be interesting to examine expected hardness based on material appearance in relations to grasp force anticipation. The current consensus is that grasp force increases with object weight (Zatsiorsky and Latash, 2008; Marneweck et al., 2016), but less is known to what extend we rely on visual material appearances during force regulations when reaching for objects made of materials that require distinct approach due to their disposition. For instance, what optical characteristics segregate perceived soft objects from perceived hard objects, and do we rely on those characteristics to anticipate the required force to successfully lift the objects? Future research will hopefully clarify these questions.

We propose that the motor control of reaching involves a mechanism that has stored associations between visual material properties and their intrinsic properties, similar to what we have for size and weight (e.g., Baugh et al., 2012). A mechanism that is constantly being updated when new information becomes available when exploring the physical world. Developmental studies on motor control have demonstrated, in fact such adaptation in reaching movements. They have shown that the control of adjustment time in reaching is an adaptive mechanism, which arises early in the perceptual motor development and is updated through repeated experiences of objects in various shapes and with various weights (Pryde et al., 1998; Berthier et al., 1999; Newman et al., 2001; Berthier and Keen, 2005; Rocha et al., 2013).

In sum, we found the temporal characteristics of the hand movements during a reaching task to be influenced by not only the weight of the cups but also by their glossy appearance and expected hardness, extending previous findings on the role of material perception in prehension. Here, we demonstrated that the deceleration period of reaching is a grasp-preparation period guided by both the expectation of the object’s weight and its glossy surface appearance. Moreover, we demonstrated that the selection of grip position before object lifting is guided by the expected material properties, in which the cups were held at their respective COM.

## Data Availability Statement

The datasets generated for this study are available on request to the corresponding author.

## Ethics Statement

Ethical review and approval was not required for the study on human participants in accordance with the local legislation and institutional requirements. The patients/participants provided their written informed consent to participate in this study.

## Author Contributions

KI developed the idea for the research, created the experimental stimuli and set up, carried out the experiment and performed all computations, and wrote the manuscript with input from CB. CB verified the analytical methods and provided theoretical and methodological discussion during the preparation, conduction, and interpretation of the experiment. Both authors discussed the results and contributed to the final manuscript.

## Conflict of Interest

The authors declare that the research was conducted in the absence of any commercial or financial relationships that could be construed as a potential conflict of interest.

## References

[B1] Adams W. J. Kerrigan I. S. Graf E. W. (2016). Touch influences perceived gloss. *Sci. Rep.* 6:21866. 10.1038/srep21866 26915492 PMC4768155

[B2] Alt N. Xu J. Steinbach E. (2015). “Grasp planning for thin-walled deformable objects,” in *Proceedings of the Robotic Hands, Grasping, and Manipulation (ICRA Workshop)*, Seattle, WA.

[B3] Bates D. M. Mächler M. Bolker B. M. Walker S. C. (2015). Fitting linear mixed-effects models using lme4. *J. Stat. Softw.* 67 1–48. 10.18637/jss.v067.i01

[B4] Battaglia P. W. Schrater P. R. (2007). Humans trade off viewing time and movement duration to improve visuomotor accuracy in a fast reaching task. *J. Neurosci.* 27 6984–6994. 10.1523/jneurosci.1309-07.2007 17596447 PMC6672223

[B5] Baugh L. A. Kao M. Johansson R. S. Flanagan J. R. (2012). Material evidence: interaction of well-learned priors and sensorimotor memory when lifting objects. *J. Neurophysiol.* 108 1262–1269. 10.1152/jn.00263.2012 22696542

[B6] Baumgartner E. Wiebel C. B. Gegenfurtner K. R. (2013). Visual and haptic representations of material properties. *Multisens. Res.* 26 429–455. 10.1163/22134808-00002429 24649528

[B7] Berthier N. E. Clifton R. K. McCall D. D. Robin D. J. (1999). Proximodistal structure of early reaching in human infants. *Exp. Brain Res.* 127 259–269. 10.1007/s002210050795 10452213

[B8] Berthier N. E. Keen R. (2005). Development of reaching in infancy. *Exp. Brain Res.* 169 507–518. 10.1007/s00221-005-0169-9 16341854

[B9] Brouwer A. M. Georgiou I. Glover S. Castiello U. (2006). Adjusting reach to lift movements to sudden visible changes in target’s weight. *Exp. Brain Res.* 173 629–636. 10.1007/s00221-006-0406-x 16525801

[B10] Buckingham G. Cant J. S. Goodale M. A. (2009). Living in a material world: how visual cues to material properties affect the way that we lift objects and perceive their weight. *J. Neurophysiol.* 102 3111–3118. 10.1152/jn.00515.2009 19793879

[B11] Buckingham G. Goodale M. A. White J. A. Westwood D. A. (2016). Equal-magnitude size-weight illusions experienced within and between object categories. *J. Vis.* 16:25. 10.1167/16.3.25 26891832

[B12] Burger B. Toiviainen P. (2013). “MoCap toolbox – a Matlab toolbox for computational analysis of movement data,” in *Proceedings of the 10th Sound and Music Computing Conference, (SMC)*, ed. Bresin R. (Stockholm: KTH Royal Institute of Technology).

[B13] Christensen R. H. B. (2018). *Ordinal –Regression Models for Ordinal Data. R Package Version 2018.8-25*. http://www.cran.r-project.org/package=ordinal/ (accessed May 07, 2019).

[B14] Cole K. J. (2008). Lifting a familiar object: visual size analysis, not memory for object weight, scales lift force. *Exp. Brain Res.* 188 551–557. 10.1007/s00221-008-1392-y 18443767

[B15] Eastough D. Edwards M. G. (2007). Movement kinematics in prehension are affected by grasping objects of different mass. *Exp. Brain Res.* 176 193–198. 10.1007/s00221-006-0749-3 17072606

[B16] Egmose I. Køppe S. (2018). Shaping of reach-to-grasp kinematics by intentions: a meta-analysis. *J. Mot. Behav.* 50 155–165. 10.1080/00222895.2017.1327407 28644719

[B17] Faul F. Erdfelder E. Lang A. G. Buchner A. (2007). G^∗^Power 3: a flexible statistical power analysis program for the social, behavioral, and biomedical sciences. *Behav. Res. Methods* 39 175–191. 10.3758/BF03193146 17695343

[B18] Fikes T. G. Klatzky R. L. Lederman S. J. (1994). Effects of object texture on precontact movement time in human prehension. *J. Mot. Behav.* 26 325–332. 10.1080/00222895.1994.9941688 12719189

[B19] Fitts P. M. (1954). The information capacity of the human motor system in controlling the amplitude of movement. *J. Exp. Psychol.* 47 381–391. 10.1037/h0055392 13174710

[B20] Flanagan J. R. Beltzner M. A. (2000). Independence of perceptual and sensorimotor predictions in the size-weight illusion. *Nat. Neurosci.* 3 737–741. 10.1038/76701 10862708

[B21] Flanagan J. R. Bittner J. P. Johansson R. S. (2008). Experience can change distinct size-weight priors engaged in lifting objects and judging their weights. *Curr. Biol.* 18 1742–1747. 10.1016/j.cub.2008.09.042 19026545

[B22] Flanagan J. R. Bowman M. C. Johansson R. S. (2006). Control strategies in object manipulation tasks. *Curr. Opin. Neurobiol.* 16 650–659. 10.1016/j.conb.2006.10.005 17084619

[B23] Flatters I. J. Otten L. Witvliet A. Henson B. Holt R. J. Culmer P. (2012). Predicting the effect of surface texture on the qualitative form of prehension. *PLoS One* 7:e32770. 10.1371/journal.pone.0032770 22403706 PMC3293844

[B24] Fleming J. Klatzky R. L. Behrmann M. (2002). Time course of planning for object and action parameters in visually guided manipulation. *Vis. Cogn.* 9 502–527. 10.1080/13506280143000557

[B25] Fleming R. W. (2014). Visual perception of materials and their properties. *Vis. Res.* 94 62–75. 10.1016/j.visres.2013.11.004 24291494

[B26] Fleming R. W. (2017). Material perception. *Annu. Rev. Vis. Sci.* 3 365–388. 10.1146/annurev-vision-102016-061429 28697677

[B27] Fleming R. W. Wiebel C. Gegenfurtner K. (2013). Perceptual qualities and material classes. *J. Vis.* 13 1–20. 10.1167/13.8.9 23847302

[B28] Freud E. Robinson A. K. Behrmann M. (2018). More than action: the dorsal pathway contributes to the perception of 3-D structure. *J. Cogn. Neurosci.* 30 1047–1058. 10.1162/jocn_a_01262 29561234

[B29] Gibson J. J. (1979). *The Ecological Approach to Visual Perception.* Boston, MA: Houghton Mifflin Harcourt.

[B30] Gordon A. M. Forssberg H. Johansson R. S. Westling G. (1991a). The integration of haptically acquired size information in the programming of precision grip. *Exp. Brain Res.* 83 483–488. 10.1007/BF00229825 2026191

[B31] Gordon A. M. Forssberg H. Johansson R. S. Westling G. (1991b). Visual size cues in the programming of manipulative forces during precision grip. *Exp. Brain Res.* 83 477–482. 10.1007/BF00230004 2026190

[B32] Grafton S. T. (2010). The cognitive neuroscience of prehension: recent developments. *Exp. Brain Res.* 204 475–491. 10.1007/s00221-010-2315-2 20532487 PMC2903689

[B33] Han G. Choi S. (2010). “Extended rate-hardness: a measure for perceived hardness,” in *Haptics: Generating and Perceiving Tangible Sensations. EuroHaptics 2010. Lecture Notes in Computer Science*, Vol. 6191 eds Kappers A. M. L. van Erp J. B. F. Bergmann Tiest W. M. van der Helm F. C. T. (Berlin: Springer).

[B34] Jeannerod M. (1981). “Intersegmental coordination during reaching at natural visual objects,” in *Attention and Performance IX*, eds Long J. Baddeley A. (Hillsdale, NJ: Lawrence Erlbaum Associates), 153–168.

[B35] Jeannerod M. (1984). The timing of natural prehension movements. *J. Mot. Behav.* 16 235–254. 10.1080/00222895.1984.10735319 15151851

[B36] Johansson R. S. (1996). “Sensory control of dexterous manipulation in humans,” in *Hand and Brain: The Neurophysiology and Psychology of Hand Movements*, eds Wing A. M. Haggard P. Flanagan J. R. (San Diego, CA: Academic Press), 381–414. 10.1016/B978-012759440-8/50025-6

[B37] Marneweck M. Lee-Miller T. Santello M. Gordon A. M. (2016). Digit position and forces covary during anticipatory control of whole-hand manipulation. *Front. Hum. Neurosci.* 10:461. 10.3389/fnhum.2016.00461 27695406 PMC5023679

[B38] Milner A. D. (2017). How do the two visual streams interact with each other? *Exp. Brain Res.* 235 1297–1308. 10.1007/s00221-017-4917-4 28255843 PMC5380689

[B39] Mon-Williams M. Murray A. H. (2000). The size of the visual size cue used for programming manipulative forces during precision grip. *Exp. Brain Res.* 135 405–410. 10.1007/s002210000538 11146818

[B40] Newman C. Atkinson J. Braddick O. (2001). The development of reaching and looking preferences in infants to objects of different sizes. *Dev. Psychol.* 37 561–572. 10.1037/0012-1649.37.4.561 11444491

[B41] Paulignan Y. Jeannerod M. MacKenzie C. Marteniuk R. (1991a). Selective perturbation of visual input during prehension movements. 2. The effects of changing object size. *Exp. Brain Res.* 87 407–420. 10.1007/BF00231858 1769391

[B42] Paulignan Y. MacKenzie C. Marteniuk R. Jeannerod M. (1991b). Selective perturbation of visual input during prehension movements 1. The effects of changing object position. *Exp. Brain Res.* 83 502–512. 10.1007/BF00229827 2026193

[B43] Paulun V. C. Gegenfurtner K. R. Goodale M. A. Fleming R. W. (2016). Effects of material properties and object orientation on precision grip kinematics. *Exp. Brain Res.* 234 2253–2265. 10.1007/s00221-016-4631-7 27016090 PMC4923101

[B44] Paulun V. C. Kleinholdermann U. Gegenfurtner K. R. Smeets J. B. J. Brenner E. (2014). Center or side: biases in selecting grasp points on small bars. *Exp. Brain Res.* 232 2061–2072. 10.1007/s00221-014-3895-z 24639066

[B45] Petrenel L. Sigaud O. Babic J. (2017). Unifying speed-accuracy trade-off and cost-benefit trade-off in human reaching movements. *Front. Hum. Neurosci.* 11:615. 10.3389/fnhum.2017.00615 29379424 PMC5770750

[B46] Pryde K. M. Roy E. A. Campbell K. (1998). Prehension in children and adults: the effects of object size. *Hum. Mov. Sci.* 17 743–752. 10.1016/S0167-9457(98)00024-4

[B47] R Core Team (2018). *R: A Language and Environment for Statistical Computing.* Vienna: R Foundation for Statistical Computing.

[B48] Rocha N. A. C. F. de Campos A. C. Silva F. P. D. S. Tudella E. (2013). Adaptive actions of young infants in the task of reaching for objects. *Dev. Psychobiol.* 55 275–282. 10.1002/dev.21026 22539262

[B49] Schubotz R. I Wurm M. F. Wittmann M. K. von Cramon D. Y. (2014). Objects tell us what action we can expect: dissociating brain areas for retrieval and exploitation of action knowledge during action observation in fMRI. *Front. Psychol.* 5:636. 10.3389/fpsyg.2014.00636 25009519 PMC4067566

[B50] Sharan L. Rosenholtz R. Adelson E. (2009). Material perception: what can you see in a brief glance? *J. Vis.* 9:784 10.1167/9.8.784

[B51] Sharan L. Rosenholtz R. Adelson E. (2014). Accuracy and speed of material categorization in real-world images. *J. Vis.* 14:12. 10.1167/14.9.12 25122216 PMC4132332

[B52] Smeets J. B. Brenner E. (1999). A new view on grasping. *Motor Control* 3 237–271. 10.1123/mcj.3.3.237 10409797

[B53] Touvet F. Roby-Brami A. Maier M. A. Eskiizmirliler S. (2014). Grasp: combined contribution of object properties and task constraints on hand and finger posture. *Exp. Brain Res.* 232 3055–3067. 10.1007/s00221-014-3990-1 24888535

[B54] Turella L. Lingnau A. (2014). Neural correlates of grasping. *Front. Hum. Neurosci.* 8:686. 10.3389/fnhum.2014.00686 25249960 PMC4158794

[B55] Weir P. L. MacKenzie C. L. Marteniuk R. G. Cargoe S. L. Frazer M. B. (1991). The effects of object weight on the kinematics of prehension. *J. Mot. Behav.* 23 192–204. 10.1080/00222895.1991.10118362 14766516

[B56] Woods A. T. Newell F. (2004). Visual, haptic and cross-modal recognition of objects and scenes. *J. Physiol. Paris* 98 147–159. 10.1016/j.jphysparis.2004.03.006 15477029

[B57] Zatsiorsky V. M. Latash M. L. (2008). Multifinger prehension: an overview. *J. Mot. Behav.* 40 446–476. 10.3200/JMBR.40.5.446-476 18782719 PMC2659677

